# Impacts of Tween-20, Glycerol, and Trehalose on Hyaluronidase Activity: Insights from Microscale Thermophoresis and Capillary Electrophoresis

**DOI:** 10.3390/molecules30194008

**Published:** 2025-10-07

**Authors:** Rouba Nasreddine, Josipa Cecić Vidoš, Alexandra Launay, Reine Nehmé

**Affiliations:** Institut de Chimie Organique et Analytique (ICOA), CNRS FR 2708—UMR 7311, Université d’Orléans, 45067 Orléans, France; rouba.nasreddine@univ-orleans.fr (R.N.); josipa.cecic-vidos@cnrs-orleans.fr (J.C.V.);

**Keywords:** additives, drug screening accuracy, bioanalytical applications, enzymatic assay design, hyaluronidase activity, inhibitor potency modulation, microscale thermophoresis, capillary electrophoresis, drug and cosmetic development

## Abstract

Additives such as surfactants (Tween-20) and cryoprotectants (glycerol and trehalose) are often used in enzymatic assays to improve the quality and long-term stabilization of proteins. However, these additives can affect the enzymatic activity and the enzyme’s affinity for active compounds, such as inhibitors, and must be considered during assay design since a slight shift in enzyme behavior may compromise the reliability of the results. In this study, the effects of Tween-20, glycerol, and trehalose on hyaluronidase (Hyal) were systematically evaluated by assessing their influence both directly—through microscale thermophoresis (MST) signals of the labeled enzyme (Hyal*)—and indirectly, by monitoring the formation of the final product of the degradation of hyaluronic acid, tetrasaccharide (Tet), using capillary electrophoresis (CE/UV). Hyal was labeled for the first time with ATTO-647 NHS ester, a commercial dye compatible with MST. Efficient labeling was achieved in a phosphate-based buffer without loss of catalytic activity. Tween-20 showed no impact on MST signals nor on enzymatic performance when used between 0.005 and 0.05% (*v*/*v*). Glycerol also did not interfere with MST measurements; however, it significantly reduced catalytic activity at concentrations above 2% (*v*/*v*). Trehalose affected Hyal* fluorescence in a concentration-dependent manner and enhanced catalytic activity even at 0.02% (*v*/*v*).

## 1. Introduction

The evaluation of the affinity between a protein and its ligand is pivotal in pharmacology and biochemistry to design new drugs and/or to understand biological processes. The dissociation constant (K_d_) of a given system provides important information on its thermodynamic properties, its specificity and selectivity, and its binding kinetics. Evaluation of K_d_ can be performed using both separative and non-separative techniques. Among separative techniques, affinity chromatography (AC) and capillary electrophoresis (CE) are powerful tools for investigating noncovalent molecular interactions and for determining dissociation or association constants of complexes [1]. Nevertheless, both approaches present challenges: in AC, immobilization of the biological targets is often problematic, whereas in CE, repeatability may be limited due to variability in injection volume precision [2]. Non-separative techniques such as isothermal titration calorimetry (ITC) [3], nuclear magnetic resonance (NMR) [4], surface plasmon resonance (SPR) [5], or a biolayer interferometer (BLI) are also widely used [6]. ITC and NMR offer the advantage of being immobilization-free techniques, but they require a large amount of highly purified proteins and ligands. In contrast, SPR and BLI rely on the immobilization of one of the studied partners, which needs special skills and can potentially alter the interactions between the components.

During the last decade, a novel technology has emerged: microscale thermophoresis (MST) [7]. It relies on the motion of molecules within a microscopic temperature gradient generated by an infrared laser (1480 nm). The local heating of samples loaded in thin capillaries at 2 to 6 °C induces thermophoretic movement of fluorescent molecules. This movement, under constant buffer conditions, depends on the state of the labeled molecule, whether it is bound or not to the ligand. The main advantage of MST is the possibility to conduct affinity studies between two partners of different sizes with a free choice of buffer (aqueous solutions or cell lysate) and without any immobilization. It enables the quantitative analysis of biomolecular interactions in solution on a microliter scale with high sensitivity (down to pM) within only a few minutes (20 min) [8,9,10,11,12,13,14].

Despite the simplicity and the versatility of MST, the buffer used to carry out the measurements, referred to as the MST buffer, typically contains additives aiming at improving the quality of the fluorescence signal of the labeled target. Among additives, surfactants, such as Tween-20, Pluronic F127, or Triton X100, are very common to avoid protein adsorption on the inner wall of MST capillaries [15,16,17]. Cryoprotectant agents such as glycerol [18] or trehalose [19,20] are often used to preserve the 3D conformational structure of proteins over time, when these latter are stored at −20 °C or −80 °C. However, trehalose and glycerol differ in their mechanisms of action: trehalose is non-penetrating and primarily stabilizes enzymes through water replacement, whereas glycerol is a penetrating agent that protects proteins directly in aqueous solution [21].

To investigate the effect of the aforementioned additives, hyaluronidase was selected (referred to as “Hyal” in this manuscript) as a model enzyme. This is the first systematic study using two complementary techniques for this purpose. Microscale thermophoresis (MST) allowed us to assess potential interactions between fluorescently labeled Hyal (Hyal*) and additives, providing insights into the quality of the scanned solutions and the interactions between the studied partners [22,23]. In parallel, capillary electrophoresis with UV detection (CE-UV) enabled evaluation of the impact of additives on the catalytic activity of Hyal by monitoring the concentration of reaction products and the inhibition potency of a given ligand [24,25]. Both the MST and CE analytical methods were previously validated by Nasreddine et al. [10] and Fayad et al. [26]; therefore, the methods were used as is, without any further developments.

Hyal was selected since it is widely used in medicine, namely, in ophthalmology, as an adjuvant to facilitate the efficacy of injected drugs. In dermatology, Hyal has been used to treat filler-related complications. For this, Hyal is an interesting, widely studied target [26,27,28]. This enzyme is responsible for the degradation of hyaluronic acid (HA). This polysaccharide is abundant in the extracellular matrix and is one of the principal components of the connective tissues, where it plays an important role in viscoelasticity and in lubricating the articulations. HA is an anionic non-sulfated glycosaminoglycan of a high molecular mass composed of repeating disaccharide units of D-glucuronic acid and N-acetyl-d-glucosamine [27,29,30,31]. The quantity and size of HA are regulated by Hyal, which cleaves the β-1,4-glycosidic bonds to produce tetrasaccharide (Tet), the main final product. Degraded HA fragments promote angiogenesis, stimulate inflammatory cytokine production, and activate signaling pathways critical to cancer progression. Hence, preserving HA from degradation has gained a lot of attention during the last decades, and numerous small natural or synthetic molecules have been reported as Hyal inhibitors [29,30,32,33]. The addition of additives in the studied media may alter not only the interaction between Hyal* and its ligand but may also change its catalytic activity.

This study aimed to establish a thorough experimental framework for the systematic and detailed investigation of the influences of various additives, with diverse functional roles, on a biological target with catalytic activity. The goal was to provide a roadmap for their rational and reliable use in protein–ligand affinity and enzyme kinetics assays, both in the absence and presence of an inhibitor of interest. Therefore, Hyal was first labeled with a commercially available fluorophore, the ATTO-647 NHS ester, for its study by MST. As far as we know, this is the first time this dye has been used for MST assays of Hyal in the red spectrum, which is why the experimental labeling conditions were first optimized. A systematic study was then conducted, where Tween-20, glycerol, and trehalose were added to the buffer used in the MST assays in the range of their usual use. The effect of these additives on the labeled and unlabeled Hyal’s activity was simultaneously investigated using CE-UV.

## 2. Results and Discussion

### 2.1. Optimization of MST Conditions and Catalytic Activity Assay for Hyal*

Since a commercial fluorophore, ATTO 647 NHS ester, was used for the first time to label hyaluronidase for MST experiments, it was essential to identify the optimal buffer conditions that would support both efficient labeling and remain compatible with subsequent MST measurements (including the addition of a surfactant in the MST buffer). In this context, three different buffers were evaluated. Phosphate–ammonium buffer was tested, as it had previously yielded favorable results with hyaluronidase when used in conjunction with the NanoTemper commercial labeling kit [10]. Carbonate buffer was included due to its favorable pH for NHS ester-mediated labeling of primary amines, which enhances conjugation efficiency [34]. Phosphate-buffered saline (PBS) was selected for its stability and widespread use in physiological studies. The concentration of the labeled enzyme and its degree of labeling (DOL) were determined using Equations (2) and (3). The results are presented in Table 1.

The recovered enzyme concentrations ranged from 6.1 to 8.5 µM, with DOL values close to 1. It is worth noting that these concentrations after labeling are estimates, which may have been slightly influenced by the multiple labeling and purification steps, as well as the sensitivity of the spectrometric method used; nonetheless, they remain sufficiently reliable for the purpose of this study.

All three buffers were suitable for labeling hyaluronidase with ATTO-647 NHS ester, with PBS standing out by providing the highest recovered concentration (8.5 µM) and a precise DOL of 1. This degree of labeling indicates that, on average, one fluorophore molecule is attached to each enzyme molecule, which is an ideal condition that helps preserve enzymatic functionality and ensures consistent thermophoretic behavior [35].

**Figure 1:**
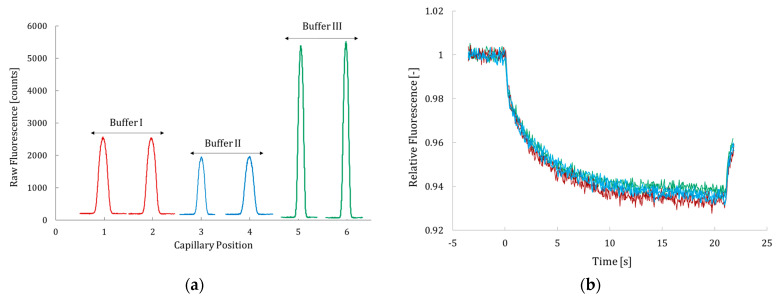
Fluorescence signal of 1 nM of Hyal* (**a**) and corresponding MST time traces (**b**). Measurements were performed using Monolith NT standard-treated capillaries at 37 °C with 40% LED excitation power. Hyal* samples were prepared by diluting the stock solution in MST buffer consisting of PBS buffer supplemented with 0.05% (*v*/*v*) Tween-20. Monolith NT.115 Pico (NanoTemper Technologies, Munich, Germany) was used. For (**a**,**b**), red traces correspond to labeling in buffer I (phosphate–ammonium buffer (pH 6.6)), blue traces correspond to that in buffer II (carbonate electrolyte solution (pH 8.2)), and green traces correspond to that in buffer III (PBS (pH 7.5)).

Then, the optimum concentration of labeled Hyal* to conduct MST experiments was determined using the apparatus pre-test mode. This optimization was based on the shape and the intensity of the provided fluorescence signal, which should be a Gaussian distribution, higher than 2000 counts when the excitation power is fixed between 20% and 60%. Additionally, the MST time trace should exhibit a smooth profile and a sufficiently high signal intensity to clearly distinguish between the bound and unbound states of the protein during affinity studies conducted in the presence of various additives or inhibitors. This test was performed on the enzyme labeled in the three different buffers, and the results are shown in Figure 1.

The results show that at 1 nM of labeled Hyal*, using a 40% excitation power, the fluorescence signal ranged from 2000 to 5500 counts under all tested labeling conditions (Figure 1a), along with smooth MST time traces (Figure 1b). An exception was observed with buffer II (carbonate buffer), where the fluorescence signal was low and close to the noise level threshold (approximately 2000 counts). In contrast, buffers I and III yielded strong fluorescence signal intensities (3500 and 5500 counts, respectively) at 1 nM of Hyal*. The fluorescence signal varied significantly across the three labeling buffers tested, with a variation of up to 40%. Despite these differences, the MST traces overlapped perfectly across all conditions, indicating that the MST buffer composition, which is composed of PBS buffer supplemented with 0.05% of Tween-20, had no observable effect on protein mobility in the MST experiments. The same observation was noted when a phosphate–ammonium buffer at pH 6.6 supplemented with 0.05% (*v*/*v*) Tween-20 was used as the MST buffer. Despite these differences between the MST buffer composition and the labeling buffer, the MST traces were perfectly superimposed under all tested conditions, suggesting that neither the MST buffer composition (phosphate–ammonium or PBS, both supplemented with 0.05% Tween-20) nor the labeling buffer had a significant impact on the thermophoretic behavior of Hyal*. Given that PBS (buffer III) yielded good labeling efficiency, a DOL of 1, and the strongest fluorescence signal, and that the MST analysis buffer shared the same base composition (PBS supplemented with 0.05% Tween-20), these conditions were considered the most straightforward and consistent. Accordingly, the enzyme labeled in PBS with the corresponding analysis buffer was used in all subsequent MST experiments. This is consistent with previous findings reported by Kharrat et al., where ATTO 647 NHS ester was successfully employed to label a small peptide, ETD151 (3 kDa), for MST studies of its interaction with liposomes [36,37]. A 172 mM PBS buffer at pH 5.5 was used throughout the entire process (for labeling and purification, and as the MST buffer), which resulted in a high labeling efficiency, the long-term stability of the labeled peptide (remaining stable for over a year), and smooth MST time traces. The results obtained in the current study for Hyal labeling with ATTO 647 NHS ester confirm the interest of this strategy, namely, maintaining the same buffer composition throughout the workflow, including labeling, purification, and MST measurements. This approach ensures assay reproducibility by minimizing buffer exchanges and reducing potential experimental artifacts.

**Figure 2:**
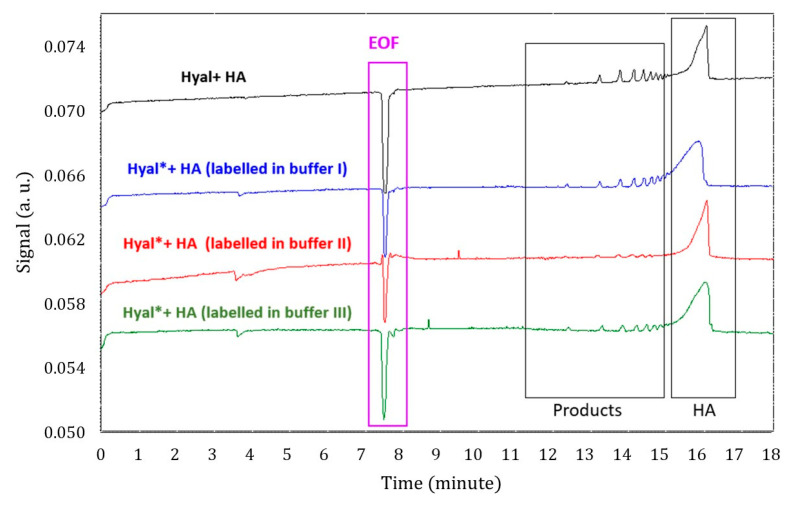
Electropherograms illustrating the degradation of hyaluronic acid (HA) by unlabeled (black) and labeled (colored) hyaluronidase and demonstrating the preservation of enzymatic activity after labeling with ATTO 647 NHS ester. The labeled enzyme was prepared using three different labeling buffers: phosphate–ammonium buffer I at pH 6.6 (blue), carbonate buffer II at pH 8.2 (red), and PBS buffer III at pH 7.5 (green). Reaction mixture composition in the incubation buffer (IB): 0.1 g/L of Hyal (labeled or unlabeled) and 0.8 g/L of HA. The incubation conditions were 180 min at 37 °C, and the IB was 2 mM sodium acetate (pH 4.0). The rinse cycles between analyses were 5 min with NaOH (1 M), 0.5 min with water, and 3 min with BGE, conducted at 30 psi (1psi = 6894.7 Pa). Electrophoretic separation conditions: BGE—50 mM ammonium acetate (pH 8.9); anodic injection—1.5 psi for 5 s; separation—+15 kV at 25 ◦C; detection—λ = 200 nm. The bare-silica capillary had a total length of 57 cm, a detection length of 47 cm, an internal diameter of 50 μm, and an outer diameter of 360.4 µm. EOF: electroosmotic flow.

Simultaneously, the catalytic activity of the labeled enzyme was evaluated and compared with that of the unlabeled enzyme. Catalytic activity was assessed by incubating the enzyme, labeled or unlabeled, with its substrate HA for 180 min at 37 °C under constant agitation (more details in Section 3 and Section 4). The results shown in Figure 2 demonstrate that the enzyme labeled in carbonate buffer was less active, as indicated by the reduced amount of degradation products in the red electropherogram. This lower activity may be attributed to the buffer’s weak buffering capacity and pH fluctuations caused by atmospheric CO_2_ dissolving into the solution over time [38]. Such pH changes could lead to enzyme precipitation during the labeling process.

Meanwhile, hyaluronidase labeled in PBS or phosphate–ammonium buffer retained its catalytic activity, as shown by the corresponding electropherograms (green and blue traces in Figure 2), which followed the same trend as that of the unlabeled enzyme (black trace). A slightly lower signal was observed for the reaction products of the labeled enzyme in these two buffers, which may reflect a modest decrease in enzymatic activity or a slightly lower actual enzyme concentration. Further investigation using more sensitive quantification methods than UV–vis spectrometry could provide valuable insights and help clarify these observations. Nevertheless, these CE-UV assays confirm that ATTO-647 NHS ester does not alter the enzyme’s catalytic site, as the labeled enzyme retained activity under all tested conditions, and the overall degradation product profile closely matched that of the unlabeled enzyme.

### 2.2. Additives’ Effects on MST Signals and Catalytic Activity of Hyaluronidase

In the following section, the effects of various additives on the fluorescence signal and thermophoretic behavior of labeled Hyal* with ATTO-647 NHS ester will be evaluated. The enzyme used for this study was labeled in PBS buffer (buffer III), and its concentration was fixed at 1 nM across all tested conditions, as described in the previous section. The MST buffer was also based on PBS supplemented with 0.05% (*v*/*v*) Tween-20, except in experiments where Tween-20 was tested as an additive, in which case, its concentration varied accordingly. In all tested conditions, solutions were loaded into a Monolith standard uncoated capillary. Simultaneously, the catalytic activity of both labeled (using buffer III, PBS, at pH 7.5) and unlabeled enzymes was assessed in the presence of different additives. Each enzymatic reaction was repeated three times, and the standard deviation of the CPA of the tetrasaccharide (Tet) product was calculated to evaluate repeatability. The presented results are the means of triplicate measurements, and error bars correspond to the standard deviations of the means of triplicate measurements. The incubation conditions followed those described in the Section 4.

#### 2.2.1. Effect of Tween-20

**Figure 3:**
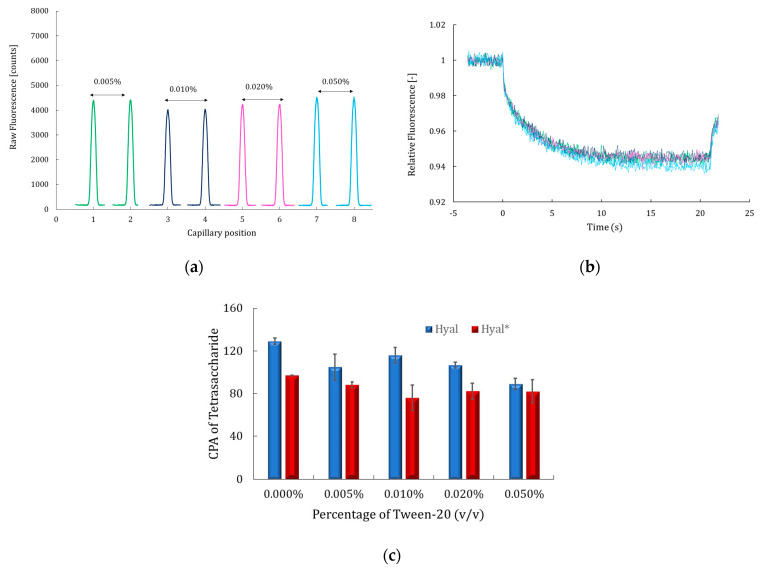
Fluorescence signal of 1 nM of labeled Hyal* (**a**) and corresponding MST time traces (**b**). For (**a**,**b**), the MST buffer consisted of PBS supplemented with different percentages (*v*/*v*) of Tween-20: 0.005% in green, 0.01% in blue, 0.02% in pink, and 0.05% in light blue. CE assay results (**c**) shown as corrected peak area (CPA) of tetrasaccharide, representing the catalytic activity of Hyal (blue histograms) and Hyal* (red histograms) in the presence of HA (0.8 g/L), incubated at 37 °C for 180 min at different percentages of Tween-20 (*v*/*v*). Each error bar represents the standard deviation (SD) of the average of three values for each tested condition. Other conditions: Figure 2.

Tween-20 was the first additive evaluated since it is added systematically to the MST buffer at 0.05% (*v*/*v*) to avoid surface adsorption, reduce aggregation, and stabilize protein structure for improved assay reliability [15,17]. Its effect on fluorescence signals and MST time traces, as well as on the enzyme catalytic activity using both labeled and unlabeled Hyal, was studied at 0.005%, 0.01%, 0.02%, and 0.05% (*v*/*v*). The results are presented in Figure 3a–c.

The results shown in Figure 3a, b indicate that Tween-20 did not affect the fluorescence signal intensity, shape, or thermophoretic movement of the labeled Hyal*. Thus, no interaction between hyaluronidase and Tween-20 was revealed in these conditions. Meanwhile, the catalytic activity of both Hyal and Hyal* was assessed by CE-UV in the presence of varying concentrations of Tween-20. As shown in Figure 3c, Tween-20 had no significant effect on enzymatic activity under any tested condition, as reflected in the red and blue histograms. Across all conditions, low error bars in the histograms confirmed the analysis’s repeatability and consistency, and variations in catalytic activity, evaluated by the CPA of the product Tet, remained below 20%, indicating a negligible impact of Tween-20. This aligns with the findings of Salameh et al. [39], who reported that the catalytic activity of lipases was also not affected by nonionic detergents like Tween-20, and it could be safely used in MST and CE assays.

#### 2.2.2. Effect of Glycerol

**Figure 4:**
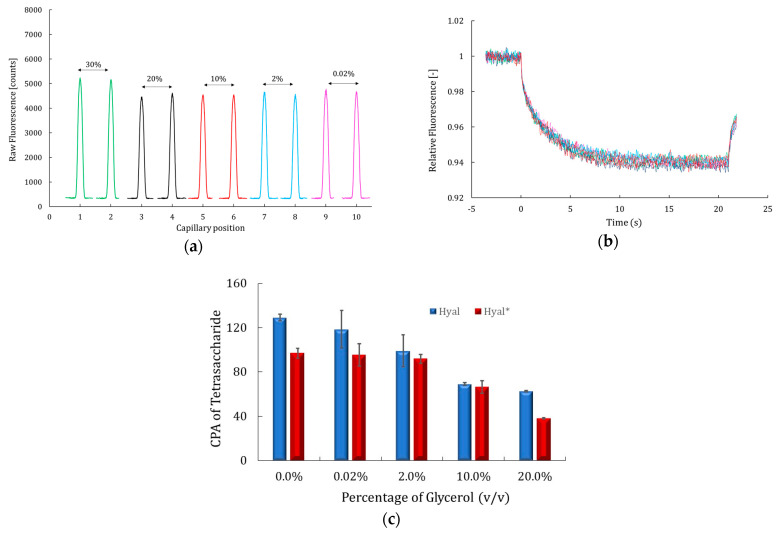
Fluorescence signal of 1 nM of labeled Hyal* (**a**) and corresponding MST time traces (**b**). For (**a**,**b**), the MST buffer consisted of PBS supplemented with 0.05% Tween-20 (*v*/*v*) and different percentages of glycerol (*v*/*v*): 0.02% in pink, 2.00% in light blue, 10.00% in red, 20.00% in black, and 30.00% in light green. CE assays results (**c**) shown as CPA of Tet representing the catalytic activity of Hyal (blue histograms) and Hyal* (red histograms) in the presence of HA (0.8 g/L), incubated at 37 °C for 180 min with different percentages of glycerol (*v*/*v*). Each error bar represents the standard deviation (SD) of the average of three values for each tested condition. Other conditions: Figure 2.

Glycerol prevents crystallization of protein during freezing, reduces its aggregation and denaturation, and maintains enzymatic/protein function over long periods of storage [18,40]. Its impact was evaluated using MST and CE-UV. The results are presented in Figure 4a–c.

As shown in Figure 4a, glycerol had only a slight effect on the fluorescence signal, with the intensity increasing by just 7% from 4800 counts at 20% (*v*/*v*) to 5200 counts at 30% (*v*/*v*). Between 0.02% and 20% (*v*/*v*), the fluorescence intensity and distribution remained stable and symmetrical, indicating a consistent signal quality. Moreover, the MST time trace recorded across all conditions perfectly overlapped (Figure 4b), demonstrating smooth, uniform profiles and no detectable interaction between glycerol and Hyal*.

In parallel, the CE-UV results demonstrated that the catalytic activity of both labeled and unlabeled hyaluronidase decreased as the glycerol concentration increased in the reaction medium, beginning at 2% (*v*/*v*) (Figure 4c). Specifically, at 10% (*v*/*v*) glycerol, the enzymatic activity was reduced by half, and at 20% (*v*/*v*), it dropped approximately threefold. Since MST analysis showed no direct interaction between glycerol and the enzyme (Figure 4a,b), this decline in enzyme activity was likely due to increased solution viscosity, which hinders substrate diffusion and, thus, reduces enzymatic efficiency.

This effect aligns with findings of G.M. Umezurike, who reported a non-linear increase in both the V_max_ and K_m_ of β-glucosidase kinetics with rising glycerol concentrations, between 3 and 16% (*v*/*v*). Because V_max_/K_m_ reflects catalytic efficiency, the observed decrease in this ratio indicates that glycerol reduces the enzyme’s efficiency, even though the apparent reaction rate increases at a high substrate concentration [41]. The same findings were obtained by Mejri et al. [42] when they investigated the effect of small carbohydrates on the catalytic activity of protease and two glycohydrolases. They found that glycerol exerted a slight inhibitory effect at 3% (*v*/*v*), while at higher concentrations, it led to a more pronounced inhibition of enzymatic activity. These results highlight the importance of carefully controlling glycerol levels in enzymatic assays to prevent unintended alterations in enzyme activity.

#### 2.2.3. Effect of Trehalose

Trehalose is a non-penetrating cryoprotectant capable of preserving the catalytic activity of enzymes by maintaining the native conformation of proteins under conditions of thermal stress, desiccation, and oxidative environments [21,43,44]. Trehalose has been classified as a kosmotrope or water structure maker, as the interaction between trehalose and water is much stronger than water–water interactions [19]. Additionally, trehalose has a high hydration ability and has the ability to substitute water around biomolecules by providing hydrogen-bonding networking, leading to maintaining the 3D structure of the active enzyme [45].

**Figure 5:**
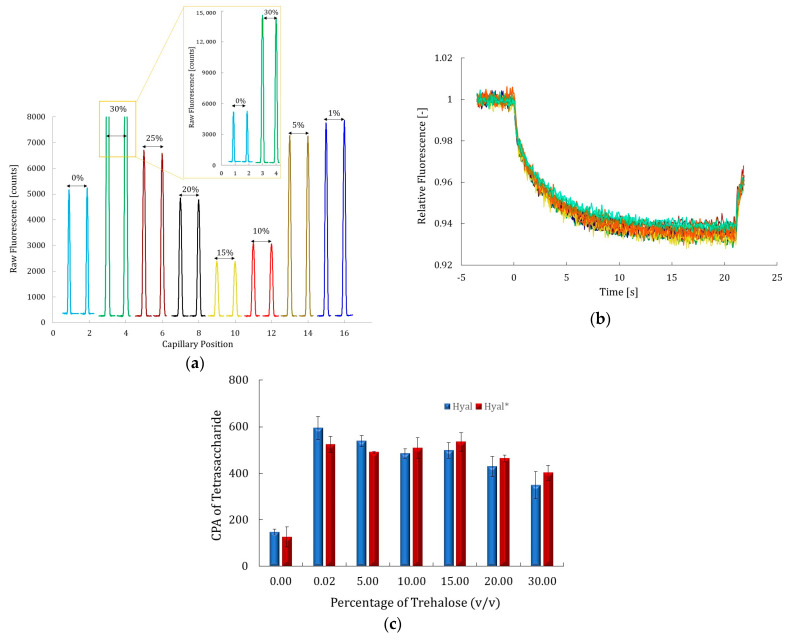
Fluorescence signal of 1 nM of labeled Hyal* (**a**) and corresponding MST time traces (**b**). For (**a**,**b**), the MST buffer consisted of PBS supplemented with 0.05% Tween-20 (*v*/*v*) and different percentages of trehalose (*v*/*v*): 0.0% in light blue, 30% in light green, 25% in brown, 20% in black, 15% in yellow, 10% in red, 5% in gold, and 1% blue. CE assay results (**c**) shown as CPA of Tet, representing the catalytic activity of Hyal (blue histograms) and Hyal* (red histograms) in the presence of HA (0.8 g/L), incubated at 37 °C for 180 min, tested with different percentages of trehalose (*v*/*v*). Other conditions: Figure 2.

Trehalose was tested at concentrations between 1% and 30% (*v*/*v*) for MST assays and between 0.02% and 30% (*v*/*v*) for CE assays; the corresponding results are depicted in Figure 5a–c.

The MST fluorescence signal intensity of Hyal* exhibited a non-monotonic dependence on the trehalose concentration. At low concentrations (1% and 5% *v*/*v*), the intensity increased by nearly 30%, rising from 5400 counts in the control sample (no trehalose) to approximately 7600 counts. However, a sharp decrease of about 50% was observed at 10% and 15% (*v*/*v*), with the intensity dropping to around 2500 counts. Interestingly, this was followed by a significant recovery: at 20% and 25% (*v*/*v*), the signal increased from 5000 to 7400 counts. The maximum intensity was recorded at 30% (*v*/*v*) trehalose, reaching 14,000 counts, approximately threefold higher than the control (Figure 5a). This non-linear behavior likely reflects a complex interplay between microenvironmental factors induced by trehalose. At low concentrations, this additive may reduce dynamic quenching by stabilizing the local environment of the fluorophore, ATTO-647 NHS ester. The fluorescence dip at intermediate concentrations could be due to transient effects on protein conformation, fluorophore accessibility, or local crowding [46].

At higher concentrations (20–30% *v*/*v*), trehalose is solubilized in the bulk water and is excluded from the solvation layer of hyaluronidase according to the preferential exclusion theory [19]. This increased viscosity of the solution likely limits molecular diffusion and dynamic quenching, while also stabilizing the ATTO-647 NHS ester, thereby reducing photobleaching and enhancing signal intensity. This result may be explained by the properties that trehalose offers at high concentrations, where it decreases the solvation layer around the protein, hence, restricting its mobility and stabilizing it in active form as the size exclusion effect becomes more pronounced. Zaroog et al. examined the effect of 1.3 M (39% *v*/*v*) of trehalose on the fluorescence signal of denatured glucoamylase [47]. They showed that the presence of trehalose increased the fluorescence signal of the protein, suggesting that the protein is more exposed to the hydrophobic clusters in the solvent, which is more likely similar to this study for the highest concentration. Meanwhile, at the lowest concentrations (10–20% *v*/*v*), an opposite effect was observed, which is supposed to be due to hindrance of the fluorescence signal, probably caused by interactions between trehalose and ATTO 647 NHS ester.

Importantly, MST traces remained perfectly superimposed under all tested conditions, indicating no detectable interaction between trehalose and labeled Hyal* (Figure 5b), and suggesting that neither the size nor the charge of the protein was altered.

In parallel, the catalytic activity of the labeled Hyal* (red histograms in Figure 5c) was evaluated by CE-UV at varying trehalose concentrations (0.02%, 10%, 15%, 20%, and 30% *v*/*v*) and compared with a trehalose-free control. The results demonstrate that this additive enhanced the enzymatic activity at all tested concentrations, with a noticeable increase even at 0.02% (*v*/*v*) that remained consistent through the 5% to 20% range. Although the activity decreased by 42% at 30% (*v*/*v*) trehalose—likely due to increased viscosity limiting substrate diffusion—it still remained approximately twice that of the control. The unlabeled enzyme followed a comparable trend, further confirming the reliability of these findings. Moreover, the small error bars in the histograms confirm the consistency and reproducibility of the results. These findings are comparable to those obtained by Stefan et al., who examined the effect of 1 M of trehalose (30% *v*/*v*) on the catalytic activity of rabbit lactate dehydrogenase toward its substrate, oxaloacetate. They demonstrated that the presence of trehalose increased its catalytic activity toward its substrate despite the fact that the viscosity was increased. This observation is consistent with the increased stability of the protein in its folded state, which limits structural fluctuations of the enzyme and thereby facilitates substrate occupancy of its subunits [48].

These complementary observations from MST and CE highlight the significant yet nuanced impact of trehalose on protein solutions. While this additive modulates the microenvironment of both the fluorophore and the enzyme, it does not compromise the enzyme integrity or its catalytic function. It can enhance protein stability and enzymatic activity under specific conditions. However, its effects are not easily predictable and may vary depending on the concentration, context, and protein’s nature, as has been shown by Uribe and Sampedro, who demonstrated that the catalytic activity of H^+^-ATPase was inhibited by the presence of trehalose, mainly due to the increase in the solution viscosity [20]. Therefore, particular attention should be given when introducing trehalose into enzymatic media—even at concentrations as low as 0.02% (*v*/*v*)—as even minimal amounts can influence assay outcomes.

### 2.3. Binding Affinity Between EGCG and Hyal* in Presence of Trehalose at 10% (v/v)

**Figure 6:**
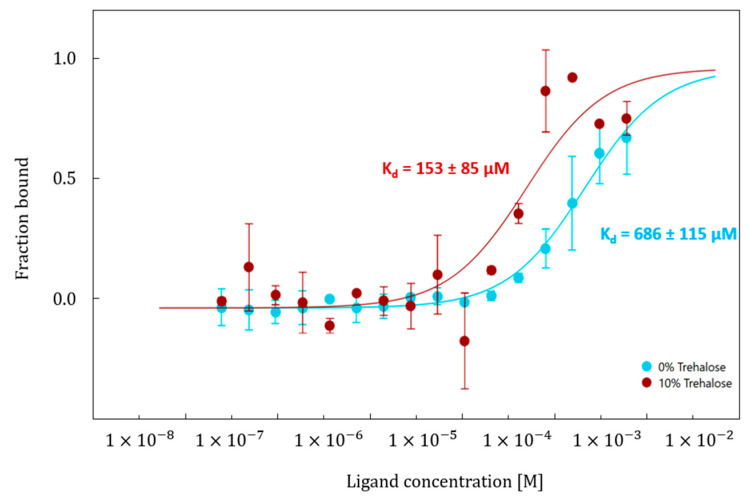
Dose–response curve of Hyal* + EGCG + 0% trehalose (light blue curve) and Hyal* + EGCG + 10% trehalose (% *v*/*v*) (red curve), fitted according to one-site binding model provided with MO affinity analysis software (v2.3). Error bars indicate the standard deviations (SDs) from two independent sets of experiments (*n* = 2). Other conditions: Section 3 and Section 4.2.

Given the pronounced and unexpectedly variable effects of trehalose, its impact on the interaction between Hyal* and the reference inhibitor EGCG was further investigated. Binding affinity assays (*n* = 2) were performed and compared with those conducted in the absence of trehalose (Figure 6). For these MST affinity measurements, 10% (*v*/*v*) trehalose was selected as a representative condition. This concentration corresponded to the lowest observed fluorescence signal in the MST analysis. If any effect on binding affinity were to be detected at this level, it would suggest that similar or stronger effects could be expected at the other concentrations. Additionally, using 10% trehalose helps minimize the influence of increased viscosity on thermophoretic movement, which becomes more pronounced at higher concentrations and could interfere with the accuracy of MST affinity measurements.

The results show that in both cases, a dose–response curve with a sigmoidal shape was obtained, featuring a well-defined lower plateau. However, the upper plateau was not fully reached due to limitations imposed by the maximum EGCG concentration used, since this latter has low water solubility. To prevent Hyal* precipitation, the concentration of organic solvents was restricted to 5% DMSO and 11% ethanol. Despite these constraints, the dissociation constant (K_d_) between EGCG and Hyal* was determined to be 686 µM in the absence of trehalose (S/N = 38), and 153 µM in the presence of 10% (*v*/*v*) trehalose (S/N = 7). The corresponding standard deviations were 115 µM and 85 µM, respectively. These results indicate that this additive impacts the interaction between EGCG and labeled Hyal* as the K_d_ value obtained in the presence of 10% (*v*/*v*) trehalose (*v*/*v*) was four times lower than the one obtained in its absence. This suggests that trehalose enhances the binding affinity between EGCG and Hyal*, likely by stabilizing the protein’s conformation in its folded state and, hence, facilitating the access to the inhibitory site by the compact inhibitor, as previously discussed [20,46,47]. A similar observation was previously reported by Nasreddine et al. [10], where the binding affinity between Hyal* and EGCG was detected only in the presence of HA in the reaction medium, further supporting the notion that this enzyme may exhibit allosteric behavior [10].

### 2.4. Evaluation of Inhibition Effect in the Presence and Absence of 10% Trehalose by CE

The inhibition effect of two inhibitors was evaluated by CE and tested only with the unlabeled enzyme. One commercial inhibitor, apigenin-7-glucoside, and one original synthesized inhibitor, a tetrasaccharide chondroitin sulfate (CS-4), were added at a constant concentration of 1 mg/mL in the reaction media in the absence and in the presence of 10% trehalose (*v*/*v*). This concentration was chosen as it represented the acceleration effect noted when this additive was added to the reaction media, as described in Figure 5c. The results are summarized in Table 2.

The results show that apigenin-7-glucoside inhibited hyaluronidase activity by 25% at 1 mg/mL in the absence of trehalose, and this inhibition was doubled in the presence of 10% (*v*/*v*) trehalose. A similar trend was observed with the original synthesized compound, CS-4, whose inhibition potency increased nearly threefold, from 32% to 91%. These findings demonstrate that this additive not only enhances the catalytic activity of the enzyme, as shown in Figure 5c, but also significantly boosts the efficacy of inhibitors.

These observations may be explained by the fact that trehalose maintains hyaluronidase in an active form by excluding the solvation layer around the protein, as described earlier [20]. This exclusion promotes the folded state of the protein and restricts its mobility, thereby preventing denaturation. At the same time, trehalose also interacts with hyaluronic acid. Recent studies have shown that trehalose acts as a stabilizing agent for HA by forming hydrogen bonds with polar residues in its structure, thereby preventing conformational changes [49]. The stabilization of both hyaluronidase and hyaluronic acid by trehalose may enhance inhibitor efficiency, as it facilitates the access of EGCG to the enzyme’s inhibitory site, which is likely allosteric in nature [10].

This dual influence underscores the importance of considering trehalose’s effects in enzymatic assays, even if its impact is difficult to foresee and may vary with concentration and molecular context.

## 3. Materials and Methods

### 3.1. Chemicals

All reagents used throughout this study were of analytical grade, and no further purification was performed before use. Ammonium acetate (CH_3_COONH_4_; purity ≥ 98%), apegenin-7-glucoside (C_21_H_20_O_10_; purity ≥ 97%), epigallocatechin gallate (EGCG; purity ≥ 95%), hyaluronidase type I-S from bovine testes (BTH; 400–1000 units/mg solid), sodium acetate (CH_3_COONa; purity ≥ 99%), sodium hydroxide (NaOH; purity ≥ 98%), oligohyaluronic acid 4 (oligo-HA4 or tetrasaccharide (Tet); C_28_H_44_N_2_O_23_), potassium dihydrogen phosphate (KH_2_PO_4_), disodium hydrogen phosphate dihydrate (Na_2_HPO_4_·2H_2_O), potassium chloride (KCl), sodium chloride (NaCl), sodium bicarbonate (NaHCO_3_), trehalose dehydrate (C_12_H_22_O_11_·2H_2_O, from *Saccharomyces cerevisiae*; purity ≥ 99%), and Tween-20 or polyethylene glycol sorbitan monolaurate were purchased from Sigma-Aldrich, Saint-Quentin Fallavier, France. Hyaluronic acid, sodium salt, *Streptococcus pyrogenes* (HA; CAS 9067-32-7—Calbiochem) was purchased from Merck Millipore, Molsheim, France. Glacial acetic acid (CH_3_COOH), ammonium hydroxide (NH_4_OH 28%), N,N-dimethylformamide (DMF, purity > 99%), and Sephadex G25 (PD Miditrap G-25, Cytiva 28-9180-08) were purchased from VWR International, Fontenay-sous-Bois, France. Glycerol (bidistilled, purity > 99.5%) was purchased from Prolabo, Fontenay-sous Bois, France. ATTO-647 NHS ester was bought from ATTO Tec- GmbH (Siegen, Germany). CS-4 (sulfated tetrasaccharides biotyloniated chrodontoin 4) was obtained as an original synthesized product from ICOA, Orléans, France [50]. Ultrapure water (18 MΩ-cm) was produced using a Merck millipore Milli-Q EQ-7000 apparatus (Merck, France). Syringes and hydrophilic polyvinylidenedifluoride (PVDF) Econo Syringe Filters, with a pore size of 0.2 μm, were purchased from Agilent, Santa Clara, CA, USA. 

### 3.2. Buffer Solutions

All buffer solutions, both with and without additives, were freshly prepared each day using ultrapure water. After adjusting the pH, they were filtered and stored at +4 °C.

#### 3.2.1. Labeling Buffers

The buffers used to carry out the labeling were freshly prepared. Three different buffers were tested, and they were prepared as follows.

Buffer I—phosphate–ammonium buffer (pH 6.6): The phosphate–ammonium buffer was prepared by dissolving the appropriate amount of NaH_2_PO_4_·2H_2_O (20 mM) and NaCl (77 mM) in ultrapure water. Then, the pH was adjusted with 1 M NH_4_OH to have a final pH of 6.6. The total ionic strength of this buffer was equal to 97 mM.

Buffer II—bicarbonate buffer (pH 8.2): Bicarbonate solution was prepared by weighing the appropriate amount of NaHCO_3_ and dissolving it in ultrapure water to obtain a concentration of 200 mM. The pH was adjusted with 1M NaOH. The total ionic strength of this buffer was equal to 200 mM.

Buffer III—PBS buffer (pH 7.5): Phosphate-buffered saline (PBS) was prepared by dissolving the appropriate amount of potassium dihydrogen phosphate (KH_2_PO_4_; 1.8 mM) and sodium phosphate dibasic (Na_2_HPO_4_·2H_2_O; 10 mM). Then, potassium chloride (KCl; 2.7 mM) and sodium chloride (NaCl; 137 mM) were added to obtain PBS at pH 7.5 in ultrapure water. The final ionic strength was equal to 172 mM.

#### 3.2.2. MST Assay Buffer

Regardless of the labeling buffer tested, the MST assays were carried out first with phosphate–ammonium buffer at pH 6.6 supplemented with 0.05% *v*/*v* Tween-20. Then, the PBS buffer supplemented with 0.05% *v*/*v* Tween-20 was tested once the best labeling buffer was selected.

#### 3.2.3. Buffers for CE Analysis

The incubation buffer (IB) for the enzymatic assay was a 2 mM solution prepared by dissolving the required amount of sodium acetate in ultrapure water. The pH was then adjusted to 4.3 with 1 M glacial acetic acid.

The background electrolyte (BGE) for electrophoretic separation was a 50 mM solution obtained by dissolving the required amount of ammonium acetate in ultrapure water. The pH was then adjusted to 8.9 with 1 M ammonium hydroxide.

### 3.3. Reagent Stock Solutions

Stock solutions of 10 mg/mL of both unlabeled BTH and HA were prepared in the filtered IB and then diluted in the same buffer to reach the required concentrations.

A stock solution of CS-A was prepared at 10 mg/mL in ultrapure water and then diluted in the IB to the appropriate concentration. Inhibitor solutions of EGCG and apigenin-7-glucoside were prepared at 5 mg/mL in a mix of organic solvents, DMSO/ethanol (30%/70%; *v*/*v*), then vortexed for 1 min, and placed in an ultrasound bath for 5 min to ensure full solubility. The prepared stock solutions were stored at +4 °C before being used in the MST and CE assays.

For BTH labeling: The enzyme was prepared at an initial concentration of 8.8 g/L (160 µM) in the tested buffer (phosphate–ammonium, carbonate, or PBS buffer) and then stored at 4 °C until use. The dye was prepared as follows: An ATTO-647 NHS ester stock solution was prepared by dissolving 70 µg of the dye with 50 µL of DMF to obtain a final concentration of 1.4 g/L.

### 3.4. Additive Stock Solutions

#### 3.4.1. Tween-20

Tween-20 was initially prepared as a 10% (*v*/*v*) solution in water. This stock was then diluted to 1% (*v*/*v*) in PBS (for MST assays) or in IB (for CE analysis) and, subsequently, added to the mixtures to obtain final concentrations of 0.005%, 0.01%, 0.02%, and 0.05% (*v*/*v*).

#### 3.4.2. Glycerol

Glycerol (≥99%) was added to both CE and MST assay mixtures by first preparing an intermediate solution of 50% (*v*/*v*). The required volume of pure glycerol was pipetted using Gilson Microman positive displacement pipettes (Villiers-Le-Bel, France) to ensure accurate handling of the viscous liquid. From this intermediate solution, appropriate aliquots were taken to achieve final glycerol concentrations of 0.2%, 2.0%, 10%, and 20% (*v*/*v*) in the reaction mixtures for the respective assays.

#### 3.4.3. Trehalose

Two separate stock preparations were prepared:

For MST assays: Trehalose was prepared as a 35% (*w*/*w*) stock solution in PBS buffer. The stock solution was homogenized using an automatic Homogenizer 850 (Fisher Scientific, Waltham, MA, USA) for seven cycles of 3 min at 11,000 rpm, with 1 min intervals between cycles to prevent overheating. The homogenizer probe measured 7 mm × 115 mm. This stock was subsequently diluted in the same buffer to obtain concentrations of 0.02%, 1%, 5%, 10%, 15%, 20%, 25%, and 30% (*v*/*v*) in the presence of the labeled enzyme.

For CE analysis: Trehalose solutions were prepared by diluting the appropriate amount of stock solution in IB to have 0.02%, 5%, 10%,15%, 20%, and 30% (*v*/*v*) in the final reaction mixture. Following homogenization (as described above), the pH was adjusted to 4.0 using 1 M acetic acid. The solutions were then filtered through a syringe filter of 0.4 µm and stored at 4 °C until used.

## 4. Instrumentation and Operating Conditions

### 4.1. Capillary Electrophoresis Analysis Conditions

(i) Off-line assays were performed to conduct enzymatic reactions following a protocol previously developed by Fayad et al. and Nasreddine et al. [26,28]. Briefly, 35 µL of the incubation buffer (with and without the additives Tween-20, glycerol, or trehalose) was pre-incubated with an appropriate volume of HA (0.8 g/L as the final concentration) for 10 min at 37 °C. Subsequently, the enzyme (labeled or unlabeled enzyme) was added to each reaction mixture, hence ensuring a constant concentration of the enzyme (labeled or unlabeled) in the mixture equal to 0.1 g/L. In all cases, the reactions were incubated at 37 °C for 180 min under constant agitation and then stopped by heating the reaction mixture in a hot water bath to 90 °C for 10 min. Each tested condition was repeated three times. The data are represented as the averages of triplicates, and the error bars represent the standard deviations (± SD). It is worth noting that all reported values presented an error of less than 20%.

(ii) For the inhibition tests, the assays were performed and stopped using the same protocol as described above. In detail, 10 µL of HA and 10 µL of the inhibitor were pre-incubated with 25 µL of IB or with 25 µL of trehalose solution before adding 5 µL of the unlabeled enzyme. PA800 plus (AB Sciex, Framingham, MA, USA) equipped with a photodiode array detector (DAD) was used to analyze the reaction mixtures controlled by the 32 Karat Software, version 9.1 (Beckman Coulter, Brea, CA, USA). The separation of charged compounds was ensured on uncoated fused silica capillaries with an inner diameter of 50 µm and a total length of 57 cm (47 cm to the detector) and detection at 200 nm (bandwidth 10 nm), purchased from Polymicro Technologies, Phoenix, AZ, USA. The samples were introduced into a preconditioned capillary from the anodic side by using the hydrodynamic mode and applying +15 kV in the normal polarity mode to ensure separation at 25 °C. Each inhibition test was repeated three times, and each value is reported as the mean ± SD %.

(iii) Before conducting analysis in the new uncoated capillaries, the latter were preconditioned with rinsing cycles of 1 M NaOH (10 min), water (10 min), and BGE (10 min). At the beginning of every working day, the capillary was first rinsed with 1 M NaOH (5 min), water (5 min), and BGE (5 min), followed by applying +15 kV (10 min). At the end of each working day, the capillary was stored overnight in water at 25 °C after being rinsed at 50 psi with NaOH (10 min) and water (10 min).

Before injecting the enzymatic assay, controls were systematically analyzed in the absence of any additives, aiming to validate the CE analytical conditions.

(iv) For quantification, the corrected peak area (CPA), which is the ratio of the area over the migration time, was used for each detected peak; in this study, the focus was on the final product, the tetrasaccharide. For inhibition assays, all inhibitors were tested at 1 mg/mL, including (i) apigenin-7-glucoside, a well-known inhibitor of hyaluronidase [26], and (ii) CS-A, the original synthesized inhibitors previously assayed for their hyaluronidase inhibition using CE [31]. In all cases, the percentage of inhibition was calculated according to Equation (1).

(1) $$\%I=\left(1-\frac{A_{x}}{A_{0}}\right)\times 100$$

The percentage of inhibition is denoted as % I. The CPA of the final product, the tetrasaccharide, in the presence of the inhibitor is denoted as A_x_, and in the absence of the inhibitor, as A_0_.

### 4.2. Microscale Thermophoresis for Hyal* Binding Assays

NT.115 Pico (NanoTemper Technologies, Munich, Germany) using standard uncoated glass capillaries was used to perform the MST assays. The excitation power was set to medium, corresponding to the LED MST power. The fluorescence wavelengths, for the excitation and emission wavelengths, were 605–645 nm and 680–685 nm, respectively. The wavelength of the laser was fixed at 1480 nm. The temperature at which the MST assays were carried out was set at 37 °C. Online MST analysis was ensured using MO.Control (version 1.6), and offline data analysis was performed with the MO.Affinity Analysis software (version 2.3). Two MST buffers were used throughout this study. The first one was phosphate–ammonium buffer at pH 6.6, which was a solution of 20 mM NaH_2_PO_4_ and 77 mM NaCl adjusted to pH 6.6 using 1 M ammonium hydroxide. The second buffer was PBS 172 mM at pH 7.5. Each MST assay was repeated twice, and all data are presented in the corresponding figures, except for trehalose, where only one set of experiments is presented.

For the binding affinity tests with EGCG in the presence and in the absence of trehalose, the results were plotted as the mean values of two sets of independent experiments (*n* = 2), and the error bars correspond to the standard deviations (SDs) from the mean values.

#### 4.2.1. Hyaluronidase Labeling

Before conducting the MST assays, hyaluronidase was chemically labeled with a commercial dye, ATTO-647 NHS-esters, following the supplier’s instructions (ATTO-TEC GMBH, Siegen, Germany) [51]. Briefly, ATTO -647 NHS ester belongs to a new generation of fluorescent tags for the red spectral region. This dye belongs to the zwitterionic family of dyes, with a net electrical charge of zero. ATTO-647 NHS ester is characterized by strong absorption, high fluorescence quantum yield, high photo-stability, and good water solubility according to the supplier’s information [51]. It binds with the amino groups of proteins by a covalent bond. All the labeling attempts were carried out at physiological pH since this fluorophore is stable in buffers up to pH 8 before being attached to a target.

The fluorophore was dissolved with DMF [34] at an initial concentration of 1.4 g/L and then incubated with the enzyme at an initial concentration of 8.8 g/L, having a protein-to-dye molar ratio equal to 1:2. The labeling was conducted overnight at 5 °C under constant agitation of 40 rotations per hour. In all tested conditions, the enzyme was prepared in the appropriate buffer at the same initial concentration of 160 µM or 8.8 g/L. Once the incubation time was over, the mixture was purified in a Sephadex G-25 gel filtration column (Cytiva, Uppsala, Sweden) of a 2 cm diameter and a 20 cm length. The cartridge used had a cutoff of 5 kDa, allowing for the removal of excess dye. Prior to purification, the cartridge was equilibrated by performing three rinsing cycles via percolation with the same buffer used for labeling. Following elution, the concentration of the collected labeled enzyme was determined using UV–visible spectrophotometry (see the following section). A total volume of 550 µL of the labeled enzyme was recovered, and 10 µL aliquots were subsequently prepared and stored at −20 °C. The activity of the labeled enzyme was verified by CE/UV analysis prior to conducting MST experiments.

#### 4.2.2. Quantification of Labeled Hyaluronidase by UV Spectrophotometry

The quantification of the labeled collected enzyme was ensured by UV spectrophotometry, as described elsewhere [10]. Briefly, a double-beam UV-1800 spectrophotometer (Shimadzu, Kyoto, Japan) with quartz cells of 0.5 mL and a 10 mm length was used to carry out the measurements. A full scan from 200 nm to 750 nm was performed to obtain the absorbance at 280 nm and 647 nm, allowing the calculation of the molar concentration of the collected labeled hyaluronidase using Equation (2) and then the DOL according to Equation (3).

(2) $$C\left(M\right)=\frac{A_{280}-\left(A_{647}\times C_{f}\right)}{{ɛ}_{280}BTH}\times FD$$

*A*_280_ denotes the absorbance of the amino acids with aromatic bonds of the protein at 280 nm; *A*_647_ denotes the corrected maximum absorbance of the ATTO-647 NHS ester dye at 647 nm. *C_f_* denotes the correction factor of the fluorophore at 280 nm (this factor is equal to 0.04 according to the supplier) and the extinction coefficient of the Hyal* at *A*_280_.

The DOL, which corresponds to the degree of labeling, indicating the amount of the dye bound to the enzyme, was calculated according to Equation (3):

(3) $$DOL=\frac{A_{647}}{ɛAtto647\times C\left(M\right)}$$

*A*_647_ is the absorbance of the collected fraction of hyaluronidase, ${ɛ}_{Atto647}=$ 1.20 × 10^5^ L·mol^−1^·cm^−1^ is the molar absorbance of the ATTO-647NHS ester dye, and C (M) is the concentration of the Hyal* calculated according to Equation (2).

#### 4.2.3. Binding Affinity Between EGCG and Hyal*

The binding affinity between the labeled Hyal* and one of its referenced inhibitors was evaluated following the same protocol described elsewhere [10]. Briefly, a stock solution of EGCG was prepared at 12 mM (5 mg/mL) in a mixed organic solvent of DMSO/ethanol 30%/70% (*v*/*v*). Then, a sixteen 1:1 serial dilution of EGCG in the same mixed organic solvent was carried out before adding the labeled enzyme Hyal* at a fixed final concentration of 1 nM. When assays were conducted in the presence of trehalose, it was added to the incubation buffer in all 16 capillary tubes at a constant concentration equivalent to 10% (*v*/*v*) before adding Hyal* at 1 nM. All solutions were then incubated for 5 min at RT and centrifuged at 10,000× *g* before being loaded into Monolith NT^TM^ standard uncoated capillaries (NanoTemper Technologies, Munich, Germany). All solutions were then scanned at 37 °C with the excitation power fixed at 40%. Dose–response curves were generated by plotting the fraction bound from the combined data of two experimental sets (*n* = 2), as a function of the ligand concentration using MO.Analysis software (version 2.3).

## 5. Conclusions

In this study, a systematic evaluation of commonly used additives was carried out using two complementary techniques: MST and CE. For the first time, Hyal was fluorescently labeled with ATTO 647 NHS ester for MST analysis. Phosphate-based buffers—specifically, phosphate–ammonium and phosphate-buffered saline (PBS)—were found to be well suited for labeling. They provided high enzyme recovery and a DOL of 1. Under the optimized conditions, as little as 1 nM of Hyal* was sufficient to generate intense, Gaussian-shaped fluorescence signals and smooth thermophoretic traces, demonstrating the dye’s compatibility with MST. The binding of the reference inhibitor EGCG to Hyal* was clearly detected, and the catalytic activity of the labeled enzyme was fully preserved, as confirmed by CE-UV.

The results revealed that Tween-20 is safe to use in the range of 0.005% to 0.05% (*v*/*v*). Glycerol did not affect MST fluorescence or thermophoretic quality up to 30% (*v*/*v*), but its use at concentrations above 2% (*v*/*v*) resulted in a marked decrease in catalytic activity. Trehalose showed, for the first time, a non-monotonic influence on MST signals across the 1–30% (*v*/*v*) range but enhanced enzymatic activity from as low as 0.02% (*v*/*v*). Additionally, 10% (*v*/*v*) trehalose improved the binding affinity to EGCG and increased the inhibition potency of other inhibitors in CE assays. Thus, its use in assay buffers should be carefully evaluated due to its variable effects on fluorescence and binding behavior. In contrast, glycerol may offer a more predictable alternative for enzyme stabilization in MST- and CE-based kinetic assays.

Together, these findings highlight the critical importance of evaluating buffer additives during assay development—considering both their chemical nature and concentration—to ensure accurate, reproducible, and physiologically relevant results. Neglecting these factors can compromise assay performance and misguide the development of effective, long-lasting bioactive compounds for cosmetic, pharmaceutical, or other biological applications. Future studies using complementary techniques such as ITC will be valuable to deepen the findings of this study and reinforce the importance of considering additives in affinity and kinetic assay design.

## Figures and Tables

**Table 1:** Hyal*’s concentration and degree of labeling (DOL), estimated by UV–visible spectroscopy according to Equations (2) and (3) (see Section 3).

	Concentration of Hyal*—Equation (2)	DOL— Equation (3)
Phosphate–ammonium (pH 6.6) (buffer I)	6.1 µM (~0.3 g/L)	1.1
Carbonate buffer (pH 8.2) (buffer II)	8.1 µM (~0.4 g/L)	1.2
PBS buffer (pH 7.5) (buffer III)	8.5 µM (~0.5 g/L)	1.0

**Table 2:** Influence of trehalose on hyaluronidase inhibition, as monitored by CE-UV. Error bars indicate the standard deviations (SDs) from three independent sets of experiments (*n* = 3). Experimental conditions: Figure 2.

Compound	Inhibition at 1 mg/mL—Equation (1)	
	0% Trehalose	+10% (*v*/*v*) Trehalose
Apigenin-7-glucoside (432 g/mol) 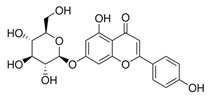	25 ± 5%	55 ± 7% (↑ × 2)
CS-4 (1406 g/mol) 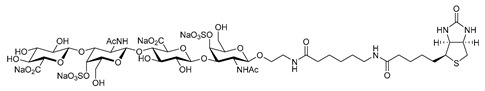	32 ± 3%	91 ± 5% (↑ × 3)

## Data Availability

The raw data supporting the conclusions of this article will be made available by the authors on request.
